# Health managers’ perspectives of community health committees’ participation in the annual health sector planning and budgeting process in a devolved unit in Kenya: a cross-sectional study

**DOI:** 10.11604/pamj.2024.47.124.40351

**Published:** 2024-03-20

**Authors:** Mildred Nanjala Wamalwa, Maximila Wanzala, Ondiek Benedict Alala

**Affiliations:** 1 Department of Public Health, Masinde Muliro University of Science and Technology, Kakamega, Kenya,; 2 Department of Accounting and Finance, Masinde Muliro University of Science and Technology, Kakamega, Kenya

**Keywords:** Community participation, community health committees, devolution, health managers, Kenya

## Abstract

**Introduction:**

health sector planning and budgeting are concerned with identifying priorities that guide budgetary allocation to improve health outcomes. Engaging the community in this process empowers them to manage their own health. Despite the benefits and the availability of legislation and structures to mainstream community participation, their involvement is minimal and marred with challenges. This study, therefore, aimed to examine the level and perspectives of health managers on community health committees´ (CHC) participation in health sector planning and budgeting.

**Methods:**

the study utilized a cross-sectional research design, incorporating both quantitative and qualitative research methods. Study participants were involved in planning and budgeting. Quantitative data were collected from 100% (n=170) of health managers, while qualitative data were gathered from 100% (n=3) of county department of health executives and 94% (n=83) of community health committee members. Descriptive statistics were utilized to analyze quantitative data, while qualitative data were analyzed thematically.

**Results:**

although 87% of the health managers agreed that community health committee participation is beneficial, only 11% of them were satisfied with their participation, and 54% rated CHC participation as low; furthermore, over 50% of health managers disagreed that Community Health Unit (CHUs) have the necessary skills to effectively participate in the process, that adequate budget and time are allocated for CHC participation, and that feedback about the process is provided to them.

**Conclusion:**

the county health department of health should allocate more funds and design sustained capacity-building programs to enhance CHC participation in health sector planning and budgeting.

## Introduction

The Alma-Ata Declaration of 1978 highlighted the integral role of community participation in the success of primary health care (PHC), asserting that community participation in setting health priorities is central to achieving universal access to PHC [1]. Five decades later, community participation is still acknowledged as pivotal in promoting people´s well-being, as reaffirmed in the Astana Declaration [2]. Moreover, a recent study emphasized that for Universal Health Coverage (UHC) and PHC to be achieved, communities need to make significant contributions [3]. Following the decentralization of the health system, communities participate formally in healthcare through community-based structures that are linked to health facilities [4]. According to the Kenya Health Community Policy 2020-2030, the operational unit for the delivery of community health services, the first tier of the health system, is a functional Community Health Unit (CHU). The Community Health Committee (CHC) serves as the governing structure of the CHU and comprises 11-13 members representing various groups within the community. Their role in planning and budgeting is to prepare and present the CHU's annual work plans and budget to the link facility health committee [5].

Analysis of case studies from Asia and Africa shows that community participation in the health sector planning and budgeting process significantly strengthens the legitimacy and accountability of the budget cycle [6]. Despite the advantages of engaging the community, a systematic review conducted on community participation in priority-setting revealed that the community was largely and consistently excluded from the process [7]. Moreover, the 2021 open budget survey showed a significant decline in global community participation in the budgeting process, with an average score of 14 out of 100 of which Kenya scored 31 out of 100 indicating limited participation [8]. Although there are structures aimed at promoting community participation in health service delivery, several challenges impede effective participation [9]. Some of the challenges include that besides the communities engaging in the process, their needs are not factored in the consolidated budget [10], inadequate structures to share information about the budget cycle with the public [11], and limited knowledge among the community members on their responsibilities concerning the budgeting process [12]. Limited research exists on the perspectives of health managers regarding CHC participation in the annual HSPB process within devolved health systems. This study aimed to examine the level of CHC participation and the perspectives of health managers on CHC participation.

## Methods

**Study setting and design:** the study was conducted in Bungoma County, one of the 47 devolved units in Kenya. The study used a mixed-methods approach, combining quantitative and qualitative methods to collect and analyze data. This approach was useful in leveraging the strengths of both methods and overcoming the shortcomings of either method when used independently.

**Study population:** the study population consisted of health managers across all levels of the county health system involved in the annual health sector planning and budgeting process. They included executive members of the county health department, county health managers, sub-county health managers, health facility managers, and community health committee members. The County health managers are responsible for strategic management and coordination of the county health services delivery. The sub-county health managers undertake the operational management within the sub-counties. The health facility managers are heads of levels 2,3 and 4 health facilities and are in charge of the day-to-day running of the facilities. To be eligible for participation in the study, the health managers needed to be involved in the annual (HSPB) process and affiliated with any level of the county health system, whereas CHC members were required to belong to a functional community health unit.

**Sample size determination:** the executive members of the county health department, county health managers, sub-county health managers, and Level 4 hospital managers were recruited using the complete enumeration method since they constitute a small fraction of the entire population of health managers. The in-charges of level 2 and 3 health facilities were determined by use of the following formula recommended by WHO for service availability and readiness assessments (SARA) for health facilities, of which health sector planning and budgeting is inclusive [13].


$$n=\left[\frac{\left[\left(z^{2}pq\right)+ME^{2}\right]}{\left[ME^{2}+Z^{2}+pq/N\right]}\right]d$$

Where: n = sample size, z = confidence level at 95% (1.96), ME = margin of error (0.15), p = the anticipated proportion of health managers with the attribute of interest (0.5), q = 1-p, N = population size and d = design effect (1.0).The sample size for Level 2 health facilities.


$$n=\left[\frac{\left[\left(1.96^{2}0.5*0.5\right)+0.15^{2}\right]}{\left[0.15^{2}+1.96^{2}+0.5*0.5/125\right]}\right]1.0=32$$

The sample size for level 3 health facilities


$$n=\left[\frac{\left[\left(1.96^{2}0.5*0.5\right)+0.15^{2}\right]}{\left[0.15^{2}+1.96^{2}+0.5*0.5/19\right]}\right]1.0=13$$

The level 2 and 3 sample sizes were increased by 10% to 35 and 15 respectively to account for anticipated non-responses [13]. Therefore, the total number of health managers recruited for quantitative data collection was 170, distributed as follows: county health managers n=10, sub-county health managers n=100, level 4 health facility managers n=10, level 3 health facility managers n=15, and level 2 health facility managers n=35. The sample size for the community health committees was determined based on the concept of data saturation, which is reached when sampling has been conducted to a point where no new viewpoints are emerging from study participants [14]. Since the CHC constitutes a homogeneous population, eight focus group discussions were adequate to achieve data saturation [15]. Each CHC consists of 11 members, and therefore 88 FGD discussants were invited to participate.

**Sampling techniques and data collection tools:** the leadership of the county health department, comprising the county executive committee member for health, chief officer of health, and the county director of health, along with the county health managers, sub-county health managers, and managers of level four facilities, were purposively recruited. The managers of health centers and dispensaries, as well as community health committees, were selected using simple random sampling. A semi-structured questionnaire, consisting of a 5-point Likert scale ranging from “1 - strongly disagree” to “5 - strongly agree,” was used to interview the health facility health managers. The county executives in the health department were interviewed as key informants, while the community health committee members participated in focus group discussions.

**Validity and reliability:** a pilot study with 17 health managers from a nearby county was undertaken to pre-test the data collection tools and the feasibility of the proposed data collection procedures. The pilot study results were also utilized to determine the reliability of the scale used to measure community engagement, which comprised eight items using Cronbach´s alpha statistic. The reliability analysis indicated that the scale had a high internal consistency, with a Cronbach's alpha (α) of 0.84, which is considered an acceptable level of internal consistency [16]. To ensure content validity for the data collection instruments, a comprehensive literature review was performed to obtain relevant items for measuring the variables.

**Data analysis:** quantitative data were analyzed using the statistical package for social sciences (SPSS v. 29.0). Descriptive analysis was performed to summarize the demographic variables using mean, standard deviation, frequencies, and percentages, while the responses on the Likert scale were analyzed using frequencies and percentages. The qualitative data was analyzed thematically.

**Ethical approval:** ethics approval was granted by the Masinde Muliro University of Science and Technology Ethics and Review Committee (MMUST/IERC/095/2022). The research license was granted by the National Council for Science and Technology (NACOSTI/P/22/19784). All the respondents signed an informed consent form before participating in the study. In addition, ethical considerations associated with data management, for instance confidentiality and safekeeping, were maintained at all stages.

## Results

**Demographic characteristics of the study participants:** the quantitative data were collected through interviews with 170 health managers, of whom 51.8% were males and 48.2% were females. The majority of the health managers (47.6%) were between the ages of 35-44, with a mean age of 42±6.76. Most of the health managers had a diploma level of education, at 48.2%. Sub-county health managers comprised the majority of those interviewed, at 58.8% (Table 1). The FGDs were conducted with 94% (n=83) of CHC members, comprising 75 females and 8 males, who were drawn from eight functional community health units. All of these units have been operational for over 10 years, having been established in 2010.

**Table 1:** demographic characteristics of the health managers

Sample characteristics	Frequency (n=170)	Percent (%)
**Sex**		
Male	88	51.8
Female	82	48.2
**Age**	**Mean age = 42±6.76**	
25-34	18	10.6
35-44	81	47.6
45-54	63	37.1
55-64	8	4.7
**Level of education**		
Diploma	82	48.2
Degree	76	44.7
Masters	11	6.5
Ph. D	1	0.6
**Category of health manager**		
County health managers	10	5.9
Sub-county health managers	100	58.8
Health facility managers	60	35.3

**Figure 1:**
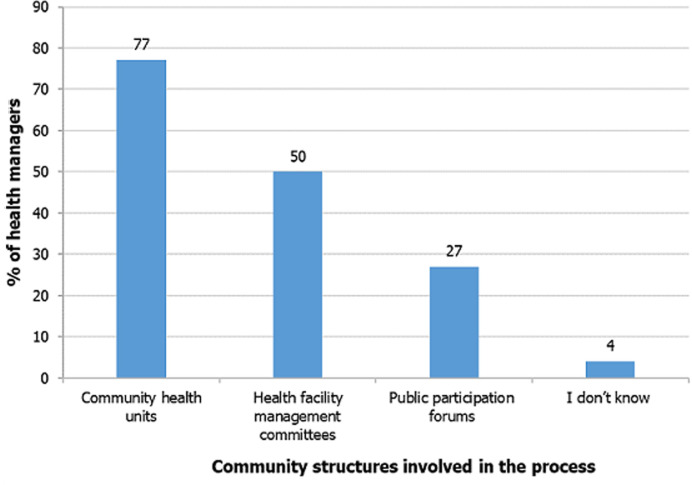
community structures involved in annual health sector planning and budgeting

**Community participation in the annual health sector planning and budgeting process:** concerning the community-level structures involved in the annual HSPB process, 77% of the health managers reported that they involved the community health units, followed by health facility management committees at 50%. Only 4% of the respondents were unaware of any community-level structures to involve in the HSPB process (Figure 1). Commenting on the community structures involved in the annual HSPB, one of the key informants said, *“Over 90% of the county is covered by functional community health units that participate in the annual planning and budgeting, their plans and budgets are submitted to the link health facilities then consolidated in the sub-county plan and budget*.” KII-3. *Another said, “The most common structure used for community engagement in the annual HSPB process is the Community Health Unit.” KII-1*.

**Figure 2:**
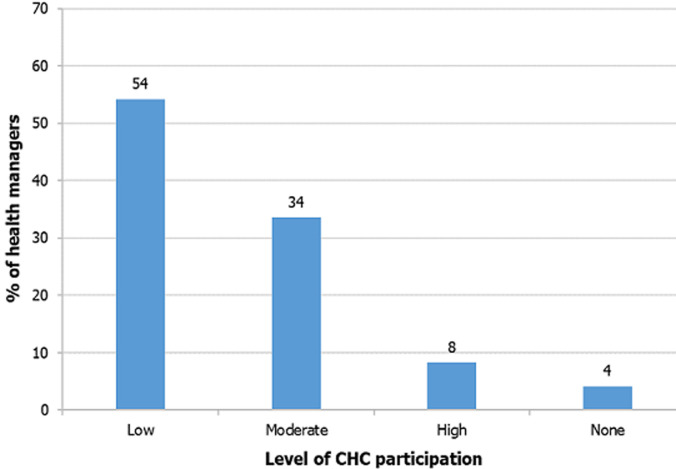
health managers' rating of the level of CHU engagement in annual health sector planning and budget process

**Level of CHC participation in the annual health sector planning and budgeting process:** fifty-four percent of the health managers rated the engagement of community health committee members in the process as low (Figure 2).

**Figure 3:**
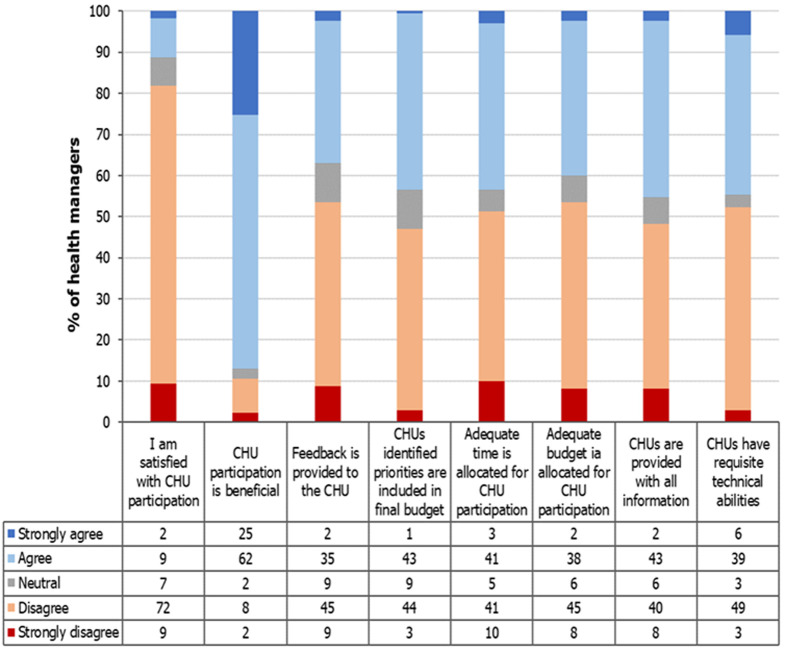
perceptions of health managers on community health units’ participation in the annual health sector planning and budgeting process

**Perspectives of the health managers on community participation:** the health manager´s perspectives on CHC engagement in the annual HSPB process were assessed using an 8-item, 5-point Likert scale whose responses ranged from 1 (strongly disagree) to 5 (strongly agree). While 87% of health managers agreed that CHC engagement is beneficial, only 11% were satisfied with their level of engagement. Over half (51%) disagreed that CHCs have the necessary skills to participate effectively. Similarly, 53% and 51% disagreed that an adequate budget and time is allocated for CHC participation, respectively. Lastly, 54% disagreed that feedback is provided to CHCs (Figure 3). The qualitative data yielded similar perspectives concerning the CHC engagement in the annual health sector planning and budgeting process, as demonstrated by the responses below: *“We are expected to participate in the development of annual plans and budgets however, we have not been trained on the annual health sector planning and budgeting process and how to complete the template.” FGD-5 “The sub-county health management team always puts a lot of pressure on us to submit a plan and budget within a few days.” FGD-7 “We never receive any feedback whatsoever from the county department of health concerning our annual work plan and budget upon submission” FGD-1 “Despite efforts to engage the CHUs throughout the planning and budgeting process, their involvement is limited due to inadequate financial resources allocated for this purpose.” KII-1 “The department's ability to organize forums for providing feedback on the process and disseminating the approved health sector plan and budget with CHUs is hindered due to insufficient financial resources.” KII-3*

## Discussion

This study aimed to assess CHC participation in the annual planning and budgeting process. The research focused on key dimensions of this participation including the level of CHC participation, the barriers they encounter, and the perspectives of the health managers concerning CHC participation. From the study, it was reported by 87% of the health managers that community participation in the annual HSPB process is essential for the success of the process. This finding supports the results of other studies which have shown that community participation in health planning and budgeting is essential because the health system serves the community and the resources used to run the health system are paid for by them therefore their participation in the process is indispensable [17]. Community participation is also beneficial in voicing community health needs [18], improving transparency and accountability [6,19] as well as promoting inclusivity, legitimacy, and acceptability of the process [20]. The engagement of established community structures for instance community health committees to foster community participation in the annual health sector planning and budgeting process is in line with those of previous studies [9,21,22]. In the wake of the decentralization of the health systems, community participation in priority setting, and the development of annual health sector plans and budgets has been given prominence leading to the establishment of community structures such as community health committees to mainstream community participation. Despite there being established community engagement structures reinforced through legislations, policy guidelines and frameworks, community participation in planning and budgeting is still low [7,9,20] consistent with the findings of this study. The 2012 open budget survey of 100 countries including Kenya, indicated that the mean score of the indicators on public engagement in the budgeting process was only 19 out of 100 [8] and it declined to 14 out of 100 in the 2021 survey [8]. Moreover, a qualitative synthesis of participation of community health committees in primary health care in sub-Saharan Africa showed that these structures are poorly engaged and often not included in the development of health facilities plans and budgets [23]. This evidence demonstrates that community participation is low and that minimal progress has been made to meaningfully engage the communities.

Contrary to this current finding, a study in Tanzania, to evaluate the findings of a social accountability program aimed at enhancing the quality of health services at primary health facilities found that 65.5% of the health facilities involved the community in annual health sector planning and budgeting [24]. This could be due to the impact of a government project focused on upgrading the ratings of public health facilities. Similarly, the findings of the open budget survey conducted in 2012 indicated that out of 100 countries surveyed, South Korea had the highest score of 92% in public participation in health sector planning and budgeting [8]. The success was attributed to heightened social and political will and the close aligning of the public engagement process with all steps of the annual budgeting cycle [25]. This may serve as a benchmark for countries struggling to make notable progress in meaningful community engagement in planning and budgeting including Kenya to learn about the extensive and innovative opportunities available to engage the community meaningfully. This study reported that the CHC members had not been trained in the annual health sector planning and budgeting process and therefore had inadequate technical capacities to meaningfully engage in the annual health sector planning and budgeting process. In line with this finding, previous studies have also shown that few of the community committee members have been trained in planning and budgeting [9,11,26,27]. The minimal technical capacities of the CHCs and their low engagement in the annual HSPB process seem to mutually reinforce each other. It has been demonstrated in the literature that due to the limited capacity of the community health committee members, health workers perceive that engaging them would not be meaningful to the process, thus their low participation [28,29]. This further aggravates the CHCs' acquisition of the relevant health sector planning and budgeting skills and experience. Considering this, it is critical to devise sustained measures aimed at building the technical capacities of the CHCs. This will enable them to participate meaningfully in the process, thereby enriching their experiences and allowing them to contribute to improved health outcomes. Further, even the low engagement of the CHCs in planning and budgeting is not without challenges. In line with the results of this study, previous studies have also reported that CHCs face several obstacles as they participate in the process. Firstly, they are allocated a limited budget and time to engage in planning and budgeting [9].

Secondly, due to limited transparency from the management, minimal information about the process is disseminated to them thus curtailing their proactive engagement in the process [10]. Finally, even after the development of their plans and budgets, the CHCs receive little to sometimes no feedback concerning the entire process, and their inputs are seldom factored in the consolidated health sector plan and budget as reported in another study in Kenya [21] and Ghana [30]. This contributes to a limited commitment from the community to participate in the process, as they find it more of a routine and not beneficial to their voiced health service delivery needs. The low engagement of community structures undermines the essence of decentralization in the health system and erodes the core principle of primary health care. These findings suggest that health sector leaders should move beyond rhetoric on community participation in health planning and budgeting as outlined in legislation, policy guidelines, and frameworks. Instead, they need to operationalize community engagement. In Kenya, this could involve disseminating and implementing guidelines for community participation, providing sustained capacity building for community health committees, increasing budgetary allocations for their engagement, and provision of feedback from health managers at all levels. These study findings support the recent call for ministries of health to strengthen community health committees through regular capacity building to enable them to effectively fulfill their roles and responsibilities [31]. The study design was cross-sectional; therefore, the findings represent the perceptions of the health managers and CHCs at a specific point in time. Although the data collected comprehensively covered health managers from all levels of the devolved health system, the generalizability of the study findings to other settings could be limited since the study was only conducted in one devolved unit.

## Conclusion

The CHCs are expected to participate in the annual planning and budgeting process by developing the CHU annual work plan and budget. However, as indicated by the findings of this study, the CHCs do not effectively fulfill this responsibility. Despite the majority of the CHUs being functional and the most involved community structure in the health sector planning and budgeting process, their level of participation is low and is marred with several barriers. To enhance CHC participation in planning and budgeting, the county health department should train the CHCs on the planning and budgeting processes. Additionally, the leadership of the county health department should allocate sufficient funds and time to enable CHCs to participate throughout all stages of the annual planning and budgeting process to ensure plans and budgets align with community needs. Finally, the county health department should conduct feedback forums with CHCs to enhance their participation in the process.

### What is known about this topic

- *Community participation plays a key role in empowering the community to enable their contribution towards improved health outcomes;*
- *Progress has been made towards enhancing community participation, including the decentralization of the health system, formulation of policies, and the enactment of laws aimed at promoting community involvement in health service delivery and management*.

### What this study adds

- *The study has emphasized the need of moving beyond mere rhetoric regarding community participation in health sector planning and budgeting, as outlined in legislation, policy guidelines, and frameworks, and instead operationalize their engagement;*
- *Effective support for community health committees is essential as they are the most commonly established community structure engaged by health managers in health sector planning and budgeting*.
